# The Impact of SARS-CoV-2 on the Most Common Comorbidities–A Retrospective Study on 814 COVID-19 Deaths in Romania

**DOI:** 10.3389/fmed.2020.567199

**Published:** 2020-09-09

**Authors:** Madalina Gabriela Barbu, Richard James Thompson, Dana Claudia Thompson, Dragos Cretoiu, Nicolae Suciu

**Affiliations:** ^1^Alessandrescu-Rusescu National Institute for Mother and Child Health, Fetal Medicine Excellence Research Center, Bucharest, Romania; ^2^Independent Researcher, Bucharest, Romania; ^3^Department of Cell and Molecular Biology and Histology, Carol Davila University of Medicine and Pharmacy, Bucharest, Romania; ^4^Division of Obstetrics, Gynecology and Neonatology, Carol Davila University of Medicine and Pharmacy, Bucharest, Romania; ^5^Alessandrescu-Rusescu National Institute for Mother and Child Health, Department of Obstetrics and Gynecology, Polizu Clinical Hospital, Bucharest, Romania

**Keywords:** coronavirus, diabetes, hypertension, chronic renal disease, coronary heart disease, cancer, stroke, COPD

## Abstract

**Background:** The SARS-CoV2 infection has rapidly spread throughout the world, particularly affecting those with underlying conditions.

**Objective:** To assess the impact of SARS-CoV-2 on the most prevalent comorbidities, among people who died of COVID-19 in Romania.

**Methods:** The study comprised 814 deaths caused by COVID-19 between 22nd March and 8th May, 2020 as reported by the Ministry of Health. WHO data regarding deaths of the general population of Romania was used for comparison. The study analyzed the demographics, number and prevalence of comorbidities and calculated the relative risk for each comorbidity.

**Results:** The study sample consisted of 61.4% males and 38.6% females; the mean age was 68.2 y; 90.9% of deaths occurred in people 50+ years. The mean number of pre-existing conditions was 2.73 (SD = 1.521), with 97.4% of the patients having at least one. The most prevalent comorbidities were hypertension (43.1%), diabetes (33.2%), and coronary heart disease (26.0%). The calculated relative risk of death due to COVID-19 was divided into 3 risk categories: high impact comorbidities (RR > 3) included diabetes RR = 6.426 (95% CI, 4.965–8.318), chronic renal disease RR = 4.338 (95% CI, 3.556–5.292) and hypertension RR=3.261 (95% CI, 2.687–3.958). The medium impact (RR = 2–3) group comprised chronic pulmonary disease RR = 2.615 (95% CI, 2.061–3.319) and chronic liver disease RR = 1.577 (95% CI, 1.183–2.104) and the low impact group (RR<2) –coronary heart disease RR = 0.664 (95% CI, 0.581–0.758), cancer RR = 0.515 (95% CI, 0.416–0.637) and stroke RR = 0.468 (95% CI, 0.370–0.593).

**Conclusion:** In the studied sample, SARS-CoV-2 had a greater impact on people with diabetes, chronic renal disease and hypertension and a lesser impact on those with coronary heart disease, cancer and stroke. Therefore, future policies in Romania should focus on shielding people in the high-risk group and prioritizing them for vaccination, whilst encouraging those in the low risk group to continue seeking treatment for their underlying diseases.

## Introduction

Coronaviruses represent a class of RNA viruses that are enveloped, positive-sense and belong to the Coronaviridae family. They can be encountered primarily in human and other mammalian hosts (1). Even though most of the human infections of coronaviruses are mild, they have been responsible for two other notable epidemics in the last two decades that had high mortality rates (10% and 37% for SARS-CoV and MERS-CoV, respectively) and caused a significant number of deaths (2–4).

The latest global pandemic reported its first pneumonia cases of unknown origin in December 2019 in the city of Wuhan, Hubei province, China (4). The virus responsible for it was identified as a novel RNA coronavirus and subsequently named SARS-CoV-2 (severe acute respiratory syndrome coronavirus 2), due to its similarities to the first SARS-CoV (5). From a genetic standpoint, SARS-Cov-2 has been found to have >80% sequence similarity with SARS-CoV and 50% with MERS-CoV (6). The most common symptoms of this condition include fever, cough, myalgia and, to a lesser extent, vomiting or nausea and diarrhea (4, 7). As of May 8, 2020, a number of 4,009,284 cases have been documented globally, with a total of 275,976 reported deaths (8). Out of the infected people, 2,337,180 were still active at that date, with 2,288,477 or 98% in a mild condition and 48,703 or 2% in a serious or critical state (8).

On the same day in Romania, there were 14,811 confirmed cases of SARS-CoV-2 infection and 922 recorded deaths caused by this pathogen (9). However, most of the patients who died had a number of comorbidities associated and, as other previous articles have suggested, some of these diseases could be directly responsible for a proportion of these deaths (10). Furthermore, other studies have also demonstrated an important correlation between certain ailments, like diabetes, and exitus due to the novel coronavirus infection, suggesting that they could represent an important underlying risk factor (11, 12). In this study, we aim to analyze the impact of SARS-CoV-2 infection on the most frequent comorbidities encountered amongst those who have died due to this pathogen in the Romanian population, and how their lifespan was influenced.

## Materials and Methods

### Patients

The first reported case of Covid-19 (Coronavirus Disease 2019) emerged in Romania on 26th February, 2020 in Gorj County, where a 20-year-old male was infected by a 71-year-old Italian man from Cattolica who was diagnosed with coronavirus. Further, on March 22nd, the first three patients died in Romania, all of whom had preexisting conditions.

The Romanian Ministry of Health centralized daily the new information received from hospitals across the country regarding the deceased patients. Furthermore, they provided a daily update, at 1 p.m., about the number of new confirmed cases, the number of people cured and the number of deaths that occurred in the past 24 h, together with a number of details regarding each deceased person. The public communication was done through the Strategic Communication Group and through the official website of the Ministry of Health at http://www.ms.ro/comunicate/. Because the data was made publicly available online, it does not require Institutional Review Board oversight and approval.

The authors took into consideration for this study all the deaths caused by SARS-CoV-2 between 22nd March and 8th May, 2020 that were reported by the Ministry of Health. The available data generally included information about the age, gender, the ward where the patient was admitted, symptoms, the date when the patient was tested, confirmed positive, the date of death and also their underlying comorbidities. All the information was anonymous, with no patient identifiers. Data regarding ethnicity or race was not included in the report, however, according to the last available census, 88.9% of the total population was represented by Romanians, 6.5% were Hungarians, 3.3% were Romani, while the rest comprised other minorities, such as Ukrainians, Germans, Turks and Russians. Information about race was not covered by this census (13). In order to be included in the study, the minimum amount of details considered essential by the authors consisted of age, gender and comorbidities (including whether they had them or not). Therefore, after manually selecting each case, out of the total number of 922 reported deaths on May 8th, only 814 met the abovementioned criteria.

From the 108 people excluded, 54.6% were from Suceava, Arad or Ialomita, where there were known outbreaks and where, because of the high number of deceased, the doctors did not always report all the details about them. Furthermore, there were a number of people that did not have a family doctor or whose records were in overseas territories, for whom it was stated that the comorbidities were not known. Finally, there were also people that have died in care homes and whose medical records were not known.

According to law number 436 from 13th March 2020, which was later updated on 6th April 2020, both being published in the Official Monitor, the protocol regarding the diagnostics of death from SARS-CoV-2 states that there are a number of situations when a doctor can write Covid-19 as the main cause on the death certificate (14, 15).

- All the patients that have died while they were hospitalized and were diagnosed with the coronavirus infection during their stay will have Covid-19 considered as their main cause of death. An autopsy will not be performed because of the infection risk for the medical personnel, unless it is done for a scientific scope or it is considered a forensic case.
- All the people that have died while in self-isolation or in quarantine upon their return from countries considered at risk will be classified as deaths due to Covid-19 and confirmed as being positive for coronavirus infection.
- All the people that have died while in hospital and were suspected to have been infected with coronavirus, but did not get a confirmation test, will be considered deaths caused by SARS-Cov-2.

The Romanian law is in accordance with the WHO International Guidelines for Certification and Classification (Coding) of Covid-19 as Cause of Death, which also states that “individual countries should not correct what is assumed to be an error, since changes at the national level will lead to data that are less comparable to data from other countries, and thus less useful for analysis.” Therefore, the authors cannot state that all of the patients who were officially reported as Covid-19 deaths by the Ministry of Health had a positive SARS-CoV-2 test, as they were not necessarily required one in order for this diagnosis to be considered on their death certificates (16).

### Terms and Measures

In this study, the authors aimed to look into the impact of the new coronavirus over certain comorbidities in a cohort of people that have died from Covid-19, how their lifespan was influenced and whether it suffered a reduction or was simply not affected. In order to achieve that desiderate, the group of Covid-19 deaths reported by the Ministry of Health was compared to the percentages of deaths caused by the studied comorbidities in the general population of Romania. However, a significant number of people that have died from the coronavirus group had multiple comorbidities, while the analysis on the general population was done by a singular cause of death.

To be able to make an appropriate comparison, a 3 stage selection criteria was put in place. First, it was taken into consideration the ward through which the patient arrived at the hospital or the train of events that led to the intake of the patient, indicating that the respective affliction was in an acute state and could have posed a potentially greater risk of exitus at that time. If the patient came through the emergency room, it was considered that for each subject in the coronavirus group, the comorbidity that would have had the highest impact on their overall life expectancy would be the one with the lowest 5 year survival rate. Therefore, a literature review was performed and a ranking system was put in place for these cases. Lastly, if the comorbidity was known for causing deaths indirectly and a 5 year life expectancy could not be found, the selection was made according to the number of reported deaths caused by it within the Romanian population. The summary of disease severity ranking considered by the authors for this study, in the absence of Covid-19, is summarized in Table 1. Further on, the 5 year survival rate is detailed for each disease.

- *Coronary Heart Diseases* (CHD) are considered the number one cause of mortality worldwide, having a particularly high impact on the Romanian population. They are responsible for over 9 million deaths per year across the globe, according to the World Health Organization (WHO) (44). Even though their mortality has progressively decreased over the last years in Western countries, thanks to the advancements in primary prevention and the improvement of diagnosis and treatment, they still represent a significant burden for developing countries (45). The 5 year survival rate after suffering an acute myocardial infarction was found to be around 56%, with 27.1% dying within a 30 day interval and 23.7% beyond that (41). Diabetes, stroke, heart failure and obesity (BMI>30) showing adjusted hazard ratios (AHR) of 1.83 (95% CI, 1.43–2.34), 1.73 (95% CI, 1.35–2.22), 1.69 (95% CI, 1.28–2.22), and 1.39 (95% CI, 1.01–1.90), respectively, were also shown to increase the mortality of CHD independent of other risk factors (41).
- *Cancer* represents the second cause of morbidity and mortality in the world. Despite the fact that Europe only represents 9% of the world population, the WHO has reported that 20.3% of the total number of deaths through cancer are encountered here (46). In Romania, cancer of all causes is responsible for ~20% of deaths, the most frequent types being lung cancer, colon cancer, breast cancer and stomach cancer (47). Some of the 5 year survival rates for the types of cancer encountered in our cohort are: for non-small cell lung cancer−61% for localized cancer, 35% for regional and 6% for distant, with an all stages combined average of 24%; for small-cell lung cancer−27% for localized cancer, 16% for regional and 3% for distant, with an all stages combined average of 6%; for colon cancer−90% for localized cancer, 71% for regional and 14% for distant, with an all stages combined average of 63%; for breast cancer−100% for Stages 0 and I, 93% for Stage II, 72% Stage III, and 22% Stage IV; for acute lymphoblastic leukemia−70% for people aged 15–24, 40% for people aged 25–64 and 15% for people over 65 years old. Because of the high number of different types of neoplasms, the rest will be summarized in Table 1.
- *Stroke* is also one of the leading causes of death around the world. In Romania, it is responsible for ~17% of all deaths (47). The 5 year survival rate for stroke was reported to be 58.3% across all types, with 59.2% of ischemic strokes surviving after 5 years, 55.4% of intracerebral hemorrhages and 55.2% of subarachnoid hemorrhages, respectively (42).
- *End Stage Renal Disease* is estimated to affect around 2 million people worldwide, with an increase of 5–7% per year. There are only two treatment options for these patients at the moment, which are transplantation or dialysis. Transplantation can be made with a kidney from a living or deceased donor and it leads to a 5 year survival rate of over 80%. However, compatible donors are difficult to find and most patients will require dialysis. This can be done in two ways—hemodialysis and peritoneal dialysis, with the vast majority (over 90%) belonging to the first group. The 5 year survival rate for patients on dialysis is 35% overall (43).
- *Chronic Pulmonary Diseases* (CPD) are represented by asthma and chronic obstructive pulmonary disease (COPD). COPD is currently the third leading cause of death around the world, with 384 million people suffering from this disease and 3 million people dying each year. At the same time, 334 million people are known to be diagnosed with asthma, therefore making it the most prevalent chronic childhood disease (48). Previous studies have shown that the 5 year survival rate for COPD after the first episode of acute exacerbation was 26% (40). In the case of asthma, the authors could not find a 5 year survival figure, therefore the severity was determined according to the number of deaths caused by asthma in Romania in one year.
- *Chronic Liver Disease* (CLD) is another global health challenge and it is defined as a hepatic suffering for more than 6 months. In this case, studies found that the 5 year survival rate was around 47%, for all causes of cirrhosis, while the average survival probabilities at 5 years were 0.66 (95% CI, 0.63–0.68) for ambulatory treated patients and 0.31 (95% CI, 0.29–0.33) following hospitalization (39).
- *Diabetes* and *Hypertension* were considered in most cases to be predictors of mortality for the abovementioned diseases. In the cases where they could not be associated with another illness, the difference between the two was made based on the number of deaths caused by each one in the Romanian population. The results lead to the conclusion that hypertension should be placed ahead of diabetes, with a number of 8,900 reported deaths caused by it in one year, against 2,700 caused by diabetes (47).

**Table 1:** Five year survival rates by disease type and subtype.

**Category**	**Disease subtype**	**5 Year survival rates**	**References**
Cancer	Mediastinum cancer–distant metastasis	0%	(17)
	Pancreas cancer stage 4	3%	(18)
	Cancer—SLC	6%	(19)
	Cancer–Leukemia (65+)	15%	(20)
	Rectal cancer—distant	15%	(21)
	Ovarian cancer–stage IV	17%	(22)
	Liver cancer	18%	(23)
	Stage 4 cervical cancer	20%	(24)
	Cancer–Breast Stage 4	22%	(25)
	Cancer—NSLC	24%	(19)
	Melanoma—distant	25%	(26)
	Gastric cancer—spread	31%	(27)
	Waldenstrom Disease—severe	36%	(28)
	Neuroendocrine tumors	39%	(29)
	Cancer–Leukemia (25–64)	40%	(20)
	Laryngeal cancer—regional	45%	(30)
	Mediastinum cancer–pulmonary metastasis	45%	(17)
	Myeloid metaplasia	50%	(31)
	Multiple myeloma—overall	52%	(32)
	Meningioma malignant	55%	(33)
	Cancer—colon	63%	(21)
	Melanoma—regional	65%	(26)
	Oropharyngeal cancer—overall	65%	(34)
	Rectal cancer—overall	67%	(21)
	Duodenal cancer—overall	68%	(35)
	Gastric cancer—localized	69%	(27)
	Cancer–Leukemia (15–24)	70%	(20)
	Chronic Leukemia over 75 years old	70%	(36)
	Meningioma benign	70%	(33)
	Rectal cancer—regional	71%	(21)
	Cancer–breast stage 3	72%	(25)
	Mediastinum cancer—localized	72%	(17)
	Meningioma atypical	75%	(33)
	Uterine cancer	75%	(37)
	Chronic Leukemia (under 75 years old)	83%	(36)
	Rectal cancer—localized	89%	(21)
	Melanoma—overall	92%	(26)
	Cancer–breast stage 2	93%	(25)
	Prostate cancer—overall	98%	(38)
	Melanoma—localized	99%	(26)
	Cancer–Breast Stage 0 & 1	100%	(25)
Chronic liver disease	Chronic Liver Disease (Hospitalized)	33%	(39)
	Chronic liver disease (ambulatory)	66%	(39)
Chronic pulmonary disease	COPD	26%	(40)
Coronary heart disease	Coronary heart disease	56%	(41)
Stroke	Stroke	58%	(42)
Chronic renal disease	Renal disease–end stage (dialysis)	35%	(43)
	Renal disease–end stage (after transplant)	80%	(43)

### Procedures

After collecting the data and categorizing it according to the abovementioned criteria, the authors proceeded to analyze it. In order to facilitate the understanding, a more visual approach was taken by creating several charts and tables. As a first step, the group of people deceased from SARS-CoV-2 was separately described.

The gender distribution was evaluated and then placed side by side with the gender distribution of deaths in the general population of Romania. Then, the age distribution was considered and also the distribution of the number of comorbidities. For these two, histograms shaped as bell curves were created, thus displaying the mean, median and mode values, together with the standard deviation. The last step was performed using the latest version of SPSS Statistics software package, developed by IBM. Further on, the cumulative number of comorbidities was calculated and converted into percentages, for both ascending and descending values and after that the prevalence of each comorbidity in the studied cohort was assessed and turned into a bar chart.

After having completed the first step and once all the data from the Covid-19 group were summarized, the study moved on to the second stage, which involved the statistical analysis of the chosen comorbidities in comparison to the deaths in the general population. The sample consisted of 814 cases which met all the necessary inclusion criteria. Out of these, 654 people had at least one of the chosen comorbidities, 22 people had no comorbidities and 137 people had other comorbidities that were not analyzed due to insufficient cases for each comorbidity to have statistical significance.

### Statistical Analysis

The second step consisted of two different statistical analyses. The first question that was answered was whether a correlation between the age and the number of comorbidities existed in the Covid-19 group. In order to achieve this, a scatter plot was created and the coefficient of determination (R square) was assessed. In this case, the R square was applied to a linear regression model that had only one independent variable (the age), therefore the following formula was used:

$$\begin{array}{c} R^{2}={\left\{\left(\frac{1}{N}\right)\times \sum \frac{\left[\left(x_{i}-\bar{x}\right)\times \left(y_{i}-\bar{y}\right)\right]}{\left(\sigma x\times \sigma y\right)}\right\}}^{2} \end{array}$$

Where *N* was the number of observations, Σ was the symbol of summation, *x*_*i*_ was the value of *x* for observation *i*, $\bar{x}$ was the mean of all the *x* values, *y*_*i*_ was the value of *y* for observation *i*, $\bar{y}$ was the mean of all the *y* values and σ*x* and σ*y* are the standard deviations of *x* and *y*, respectively (49).

The next question was whether the novel coronavirus impacted certain comorbidities and by how much was the risk of death increased for the people infected, compared to the number of deaths caused by these diseases under normal circumstances. To answer this, the authors first applied the Pearson's chi-squared test to assess if in the sets of categorical variables used, any observed differences between them was due to chance. The mathematical formula for this was:

$$\begin{array}{c} x_{c}^{2}=\sum \frac{{\left(O_{i}-E_{i}\right)}^{2}}{E_{i}} \end{array}$$

Where c represented the degrees of freedom, O were the observed values and E were the expected values. A value of *p* ≤ 0.05 was indicative of statistical significance.

Further on, in order to quantify the effect of SARS-CoV-2 over the risk of death by having certain comorbidities associated, the authors computed the relative risk (RR), the odds ratio (OR), the attributable risk (AR), and the attributable risk percent (AR%). For all of their values, a 95% confidence interval (CI) was considered, and the lower and upper limits of the interval were presented. The formulae were calculated given the following example of a contingency table (Table 2):

$$\begin{array}{c} Relative\;Risk\;\left(RR\right)=\frac{\left(\frac{a}{a+b}\right)}{\left(\frac{c}{c+d}\right)}\\ Odds\;Ratio\;\left(OR\right)=\frac{\frac{a}{b}}{\frac{c}{d}}\\ Attributable\;Risk\;\left(AR\right)=\left(\frac{a}{a+b}\right)-\left(\frac{c}{c+d}\right)\\ Attributable\;Risk\;Percent\;\left(AR\%\right)=\frac{\left(\frac{a}{a+b}\right)-\left(\frac{c}{c+d}\right)}{\left(\frac{a}{a+b}\right)}\times 100 \end{array}$$

The statistical analysis was conducted using IBM SPSS Statistics for Windows, Version 26.0 (IBM Corp., Armonk, NY, USA).

**Table 2:** Contingency table to showcase the formulae for RR, OR, AR, and AR%.

**Factors**	**Comorbidity**		**Total**
	**Yes**	**No**	
SARS-CoV-2 exposed group	A	B	a+b
SARS-CoV-2 non-exposed group	C	D	c+d

## Results

The total number of reported deaths due to SARS-CoV-2 infection until the 8th of May was 922, out of which 13 were excluded because the data did not contain age and/or gender information. Further, 95 reported cases lacked information about preexisting comorbidities, leading to a sample size of 814 patients, which was analyzed for age, gender and the number of comorbidities distribution. Out of this sample, 21 patients had no preexisting conditions and 139 had various others that were not included in the study because the preliminary data did not result in significantly statistical information, meaning that the sample analyzed for the prevalence of comorbidities consisted of 654 cases (Table 3). Most of the excluded cases for lack of information pertained to highly infected areas where probably not enough time was available for thorough anamnesis and/or data uploading.

**Table 3:** Sample sizes and exclusion criteria.

**Sample category**	**Frequency**	**Percent**	**Mean age**	**Mean no. of comorbidities**	**% Male**
Total deaths in Romania	922	100%	–	–	–
Total with age & gender info	**909**	**98.6%**	**68.1**	–	**61.4%**
(Those without age & gender)	13	1.4%	66.5	–	**–**
Total with comorbidity info listed*	**814**	**88.3%**	**68.3**	**2.73**	**61.4%**
(Those without comorbidity info listed)	95	10.3%	66.7	–	61.1%
Total with studied comorbidities**	**654**	**70.9%**	**68.7**	**2.87**	**61.6%**
(Those with other comorbidities)	139	15.1%	67.7	2.52	58.3%
(Those recorded with no comorbidities)	21	2.3%	59.0	–	76.2%

* *This figure was used for all the analyses except prevalence*.

** *Total used for the study of disease prevalence*.

### Sample Data

**Figure 1:**
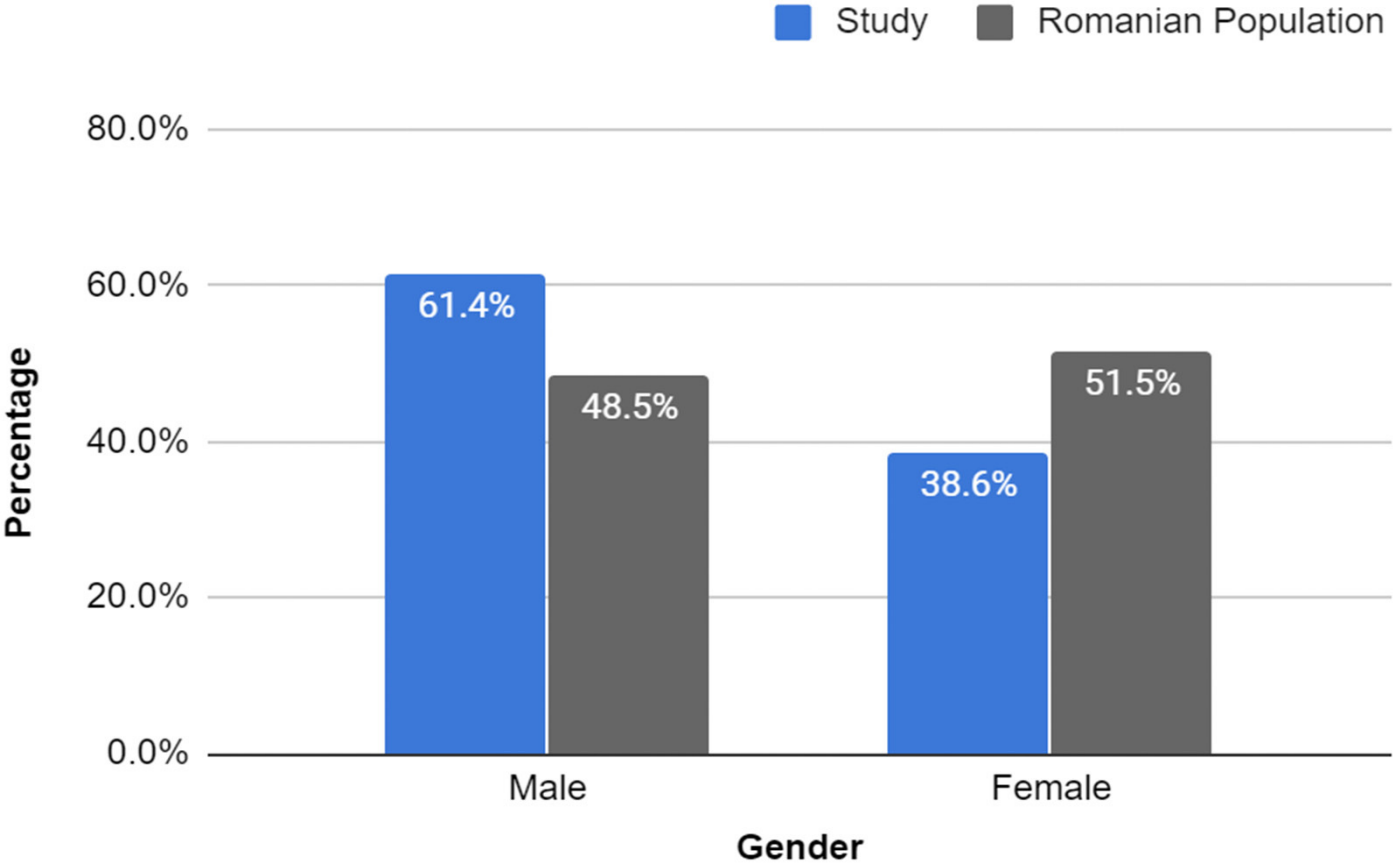
Gender percentage of study sample vs. Romanian population. The percentages of male and female deaths were clearly different from those of the general population. Study: Male (500): 61.4%, Female (314): 38.6% vs. Romanian Population: 48.5 and 51.5%, respectively. Male RR = 1.268 (95% CI, 1.200–1.339), *p* < 0.0001; Female RR = 0.748 (95% CI, 0.686–0.816), *p* < 0.0001. Source of Romanian Population Deaths: WHO Member States 2016 (47).

After the exclusion criteria detailed above were applied, the COVID-19 study group consisted of 814 patients that were reported to have died because of SARS-CoV-2 infection between 22nd March and 8th May 2020, out of which 500 were males (61.4%) and 314 were females (38.6%) (Figure 1).

Further, we compared the obtained results with the most recent statistical data of the general Romanian population deaths provided by the WHO, which showed a slightly higher percentage of female deaths (51.5 vs. 48.5%) in the year 2016. Thus, the relative risk of each gender dying due to COVID-19 was calculated and it was discovered that males had an RR of 1.27 (95% CI, 1.200–1.339), *p* < 0.0001, meaning that they had a 27% increase in the risk of death in the event of SARS-CoV-2 infection, while females had a RR of 0.75 (95% CI, 0.686–0.816), *p* < 0.0001, translating into a 25% decrease in the risk of COVID-19 exitus.

**Figure 2:**
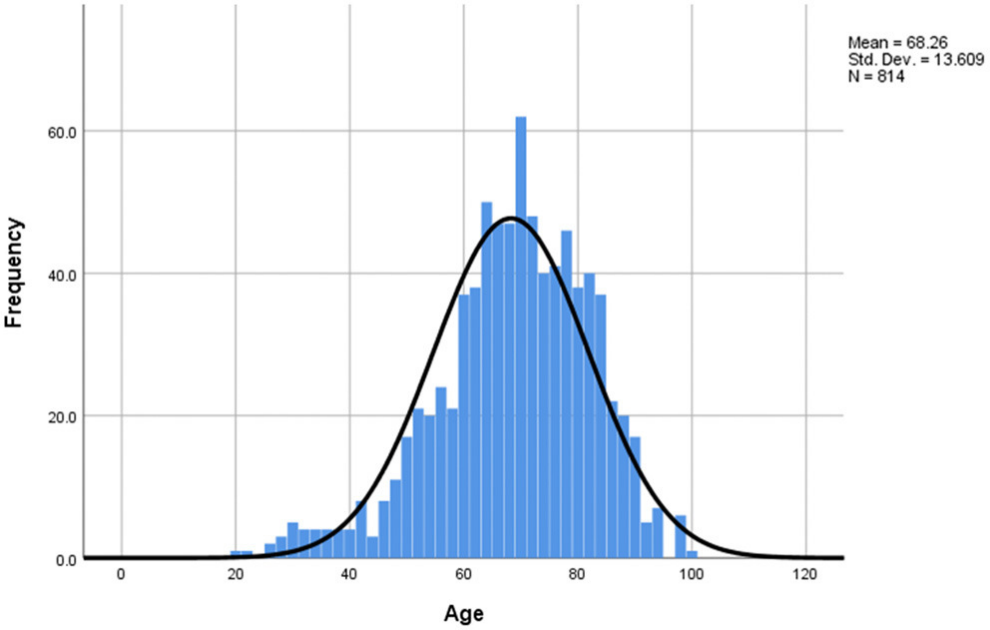
Age distribution of study sample. The mean age of death = 68.26 y, median = 69 y, mode = 70 y, min = 20 y, max = 98 y, SD = 13.609. Distribution by age intervals: 0–19: 0%, 20–29: 0.9%, 30–39: 2.9%, 40–49: 5.3%, 50–59: 13.3%, 60–69: 28.0%, 70–79: 28.3%, 80–89: 18.1%, 90–99: 3.3%. 77.6% of deaths were over 60 (22.4% under) and 90.9% of deaths were 50+ (9.1% under 50).

The age distribution of the COVID-19 study group was analyzed and it was observed that the mean age of death due to SARS-CoV-2 infection was 68.26 y (SD 13.609) (Figure 2), being slightly higher for females (70.2 y) compared to males (67.1 y). Out of the 814 patients studied, 740 deaths (90.9%) occurred to those over 50 y. The percentages for each age group can be found in the description of Figure 2 below.

**Figure 3:**
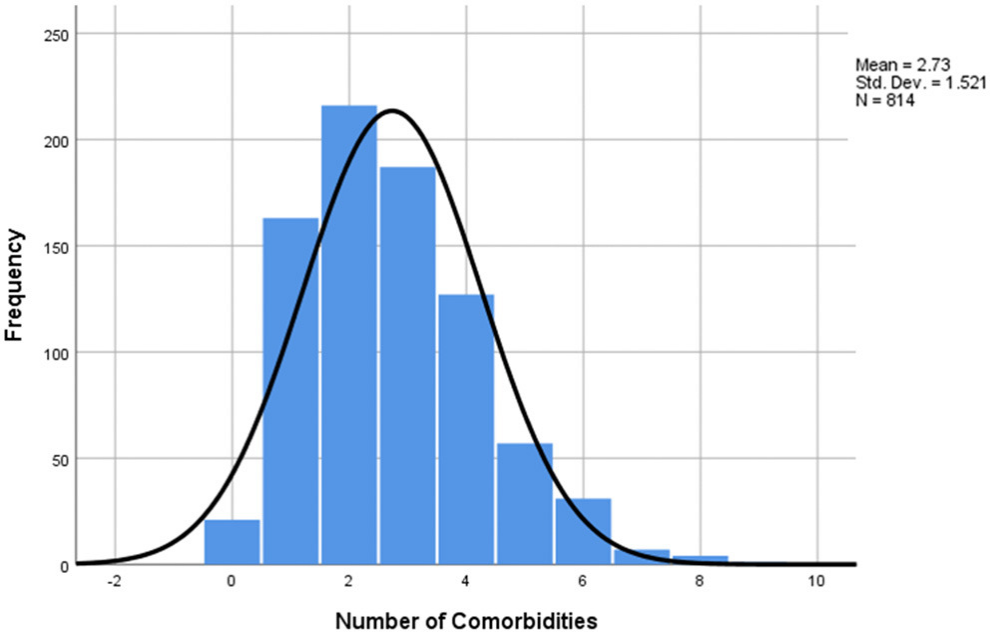
Number of comorbidities distribution of study sample. The mean number of comorbidities was 2.73, median = 3, mode = 2, min =0 (21 patients), max =9 (1 patient), SD = 1.51.

In the next step, the distribution of the number of comorbidities was taken into consideration, and it was found that the mean number of underlying diseases was 2.73 (SD=1.521) (Figure 3), with one patient having nine comorbidities reported and 21 patients having none. Furthermore, the cumulative frequency by number of comorbidities was calculated and the data obtained showed that 793 patients (97.4%) had at least one underlying ailment, while 415 patients (50.9%) had three or more (Table 4). Additional data on the cumulative frequency of comorbidities can be found in Table 4.

**Table 4:** Cumulative frequency by number of comorbidities of study sample.

**No. of comorbidities**	**Frequency**	**Percent**	**Cumulative percent**	**Inverted cumulative**
0	21	2.6%	2.6%	100.0%
1	163	20.0%	22.6%	97.4%
2	215	26.4%	49.0%	77.4%
3	187	23.0%	72.0%	50.9%
4	127	15.6%	87.6%	27.9%
5	57	7.0%	94.6%	12.3%
6	31	3.8%	98.4%	5.3%
7	8	0.9%	99.3%	1.5%
8	4	0.5%	99.8%	0.6%
9	1	0.1%	99.9%	0.1%
**Total**	**814**	**100.0%**	**100.0%**	**100.0%**

**Figure 4:**
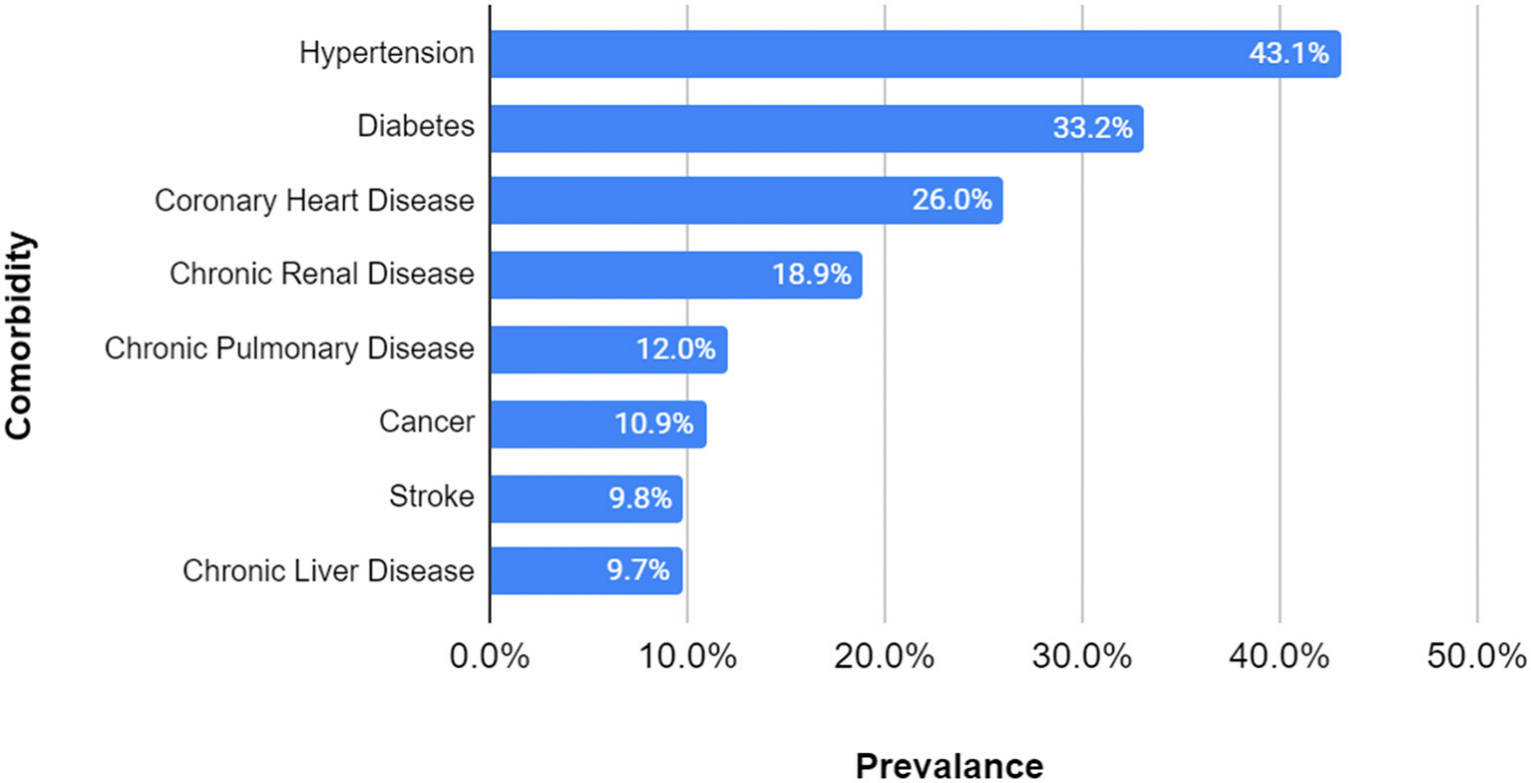
Comorbidity prevalence in study group. Sample size = 654 (108 excluded for lack of information, 21 had no comorbidities, and 139 had other comorbidities). The most prevalent three comorbidities were hypertension, diabetes and coronary heart disease, while cancer, stroke, and chronic liver disease were the least prevalent.

As mentioned in the beginning of the Results section, out of the total of 814 cases studied, 21 more patients were excluded for the comorbidity prevalence analysis because they had no preexisting condition and 139 had other comorbidities than those studied, leading to a sample size, for this particular analysis only, of 654 cases. Hypertension (43.1%), diabetes (33.2%), and coronary heart disease (26.0%) were the three most prevalent preexisting conditions among the patients that died due to SARS-CoV-2 infection, while cancer (10.9%), stroke (9.8%) and chronic liver disease (9.7%) were the least prevalent (Figure 4).

Further, after applying the criteria for the most severe comorbidity, the prevalence of comorbidities in the study group, which included those without preexisting conditions and patients with other diseases (814 cases), was compared to the mortality data provided by the WHO for the general population of Romania. As can be seen in Table 5, some of the comorbidities, such as hypertension (11.3 vs. 3.5%), chronic renal disease (10.8 vs. 2.5%), chronic pulmonary disease (7.7 vs. 3.0%) and diabetes (6.8 vs. 1.1%) had higher frequencies in the COVID-19 group than in the general population. In contrast, coronary heart disease (21.0 vs. 31.7%), cancer (9.5 vs. 18.4%) and stroke (7.9 vs. 16.8%) were less frequent in the study group than in the general population (Table 5). Small differences were observed in the percentages of those with no comorbidities (2.6 vs. 3.8%) and those that had other conditions (17.1 vs. 16.0%). For comparison, the authors further included a table comprising 10 other relevant countries by region, showcasing the difference in prevalence amongst the people that have died of these particular diseases (Table 6). The countries were selected taking into consideration the quality of the reported data, as per the WHO assessment (47). The table shows that Romania fits into the central and eastern European model, having similar figures to Russia, with higher than average rates for coronary heart diseases and stroke and a lower incidence of diabetes and chronic pulmonary disease. To ascertain the relative risk of each comorbidity by country, a further analysis should be done within that country's Covid-19 data set.

**Table 5:** Death frequency by comorbidity for study group and Romanian population.

**Most severe comorbidity**	**Study sample**		**Romanian population**	
	**Frequency**	**%**	**Deaths ('000)**	**%**
Coronary heart disease	171	21.0%	81.3	31.7%
Hypertension	92	11.3%	8.9	3.5%
Chronic renal disease	88	10.8%	6.4	2.5%
Cancer	77	9.5%	47.2	18.4%
Stroke	64	7.9%	43.1	16.8%
Chronic pulmonary disease	63	7.7%	7.6	3.0%
Diabetes	55	6.8%	2.7	1.1%
Chronic liver disease	44	5.4%	8.8	3.4%
No comorbidities*	21	2.6%	9.8	3.8%
Other comorbidities	139	17.1%	41.0	16.0%
Total**	814	100.0%	256.8	100.0%

* *This included injuries and substance abuse*.

** *500 neonatal and maternal deaths were excluded from the data set for the Romanian population, as they were not relevant for this study*.

*Source of Romanian Population Deaths: WHO Member States 2016 (47)*.

**Table 6:** Death frequency by comorbidity for Romania vs. ten other countries.

**Region**	**World avg**.	**Romania**	**Australia**	**Brazil**	**China**	**Israel**	**Italy**	**Japan**	**Russia**	**South Africa**	**United Kingdom**	**USA**
	**%**	**%**	**%**	**%**	**%**	**%**	**%**	**%**	**%**	**%**	**%**	**%**
Coronary heart disease	17.4	31.7	14.9	13.2	18.7	11.7	17.6	11.7	32.1	8.2	12.8	17.9
Cancer*	15.0	18.4	26.2	16.3	21.7	24.8	24.4	26.9	17.4	9.3	25.6	20.5
Stroke	10.7	16.8	6.8	8.6	19.6	5.6	9.7	9.6	16.5	6.0	6.4	5.3
Chronic pulmonary disease**	6.4	3.0	5.9	5.0	8.9	4.5	5.0	5.2	1.7	4.2	6.5	7.1
Diabetes	2.9	1.1	3.0	5.0	1.6	6.1	3.5	1.1	0.5	7.3	1.1	3.0
Chronic liver disease	2.3	3.4	1.0	1.9	1.5	0.9	1.4	1.3	2.2	1.1	1.3	1.8
Chronic renal disease	2.2	2.5	1.6	2.0	1.9	4.1	2.3	2.7	0.5	2.2	0.8	2.4
Hypertension	1.7	3.5	0.8	1.6	2.7	1.4	2.8	0.3	1.1	2.4	0.6	1.7
Other comorbidities	32.2	16.0	33.0	33.8	16.1	36.6	29.6	36.3	19.1	49.5	41.0	32.4
No comorbidities***	9.3	3.8	6.7	12.5	7.3	4.3	3.9	4.9	8.8	9.6	3.9	7.9

* *Includes cancers present among patients in this study, as listed in Table 1. Other cancers are included in Other Comorbidities*.

** *Includes COPD and Asthma*.

*** *Includes deaths from injury and substance abuse*.

*Neonatal and maternal deaths have been excluded for the total deaths, as they are not relevant for this study*.

*Countries were selected in each world region, to give a diverse range, based on a balance of the quality of data as determined by the WHO, population size and covid-19 deaths*.

*The world average includes countries with good and poor quality data, as judged by the WHO, so should be considered with caution*.

*Source: WHO Member States 2016 (47)*.

Obesity was also a potential risk factor but was not included as part of the studied comorbidities, as it was classified together with other metabolic disorders as a cause of death in the WHO reference data. 137 patients (16.8% of 814) had obesity listed on their death certificates in total and 37 patients had obesity in the other comorbidities group (12.2% of 139). The average age of death for those with obesity was 58.8 y, while those with normal weight had an average age of 70.2 y. Obesity was present in 40.5% of cases for those under 50 y and in 14.5% for those over 50 y.

### Statistical Analysis

**Figure 5:**
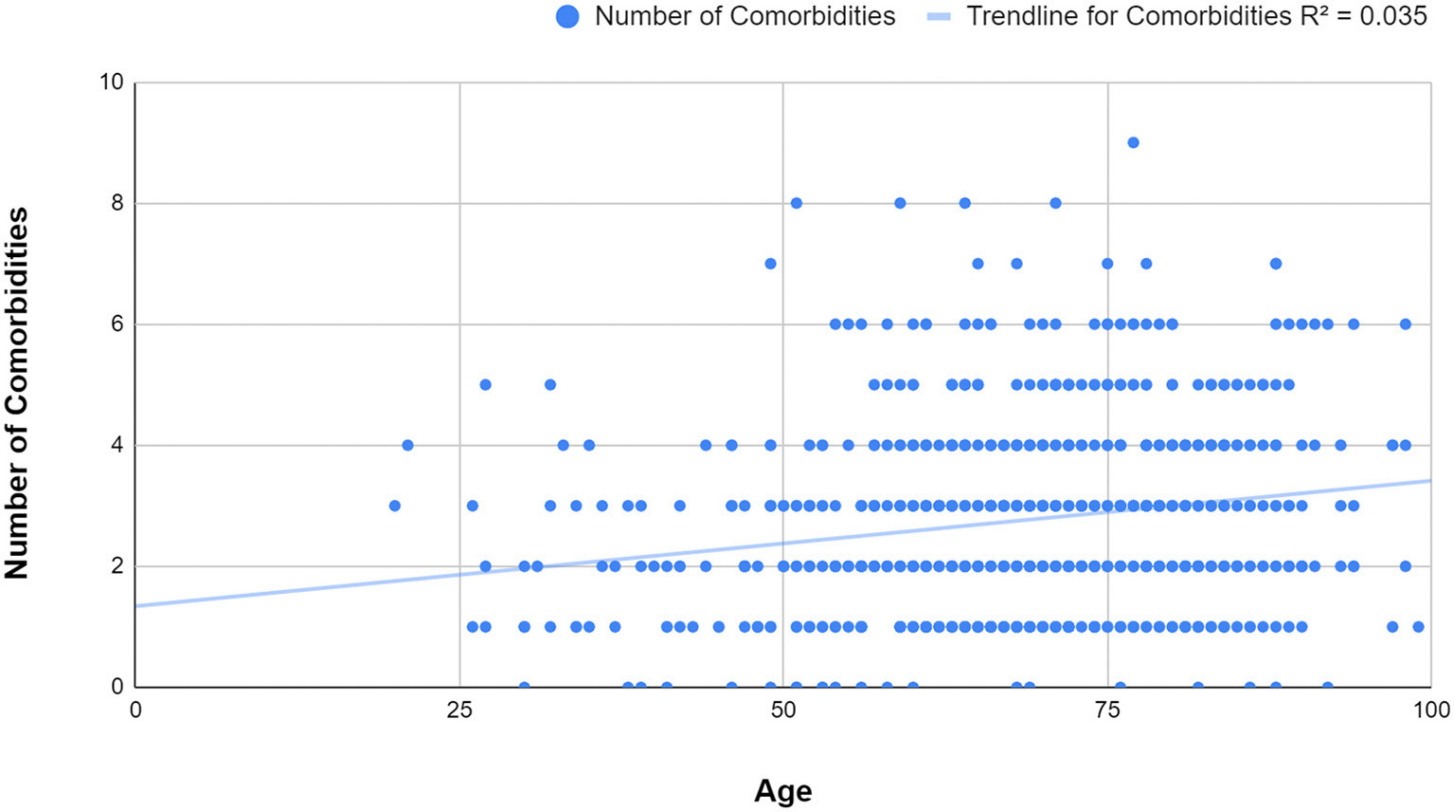
Figure 5 Linear regression scatter plot of age and number of comorbidities. It can be observed that the number of comorbidities slightly increases with age (R squared = 0.035).

In order to assess the relationship between the number of comorbidities and age, a linear regression model was applied, illustrated in Figure 5. The findings showed that the number of comorbidities did increase with age, however the R squared value indicated to us that age was not a major influencing factor for the number of underlying diseases. In this case, the R squared value was 0.035, meaning that in only 3.5% of cases the correlation between age and comorbidities was positive. The overall mean number of comorbidities was 2.73 (SD=1.521), with a slightly lower average observed in younger ages (39, 40, 42, 43, 46–51) than in older patients (80–89)−2.7 vs. 3.0 (Table 7).

**Table 7:** Mean number of comorbidities by age.

**Age**	**Frequency**	**Mean No. comorbidities**	**Standard deviation**
0–19	0.0%	0.0	0.0
20–29	0.9%	2.71	1.50
30–39	2.9%	1.92	1.35
40–49	5.3%	2.12	1.37
50–59	13.3%	2.38	1.55
60–69	28.0%	2.69	1.46
70–79	28.3%	2.93	1.54
80–89	18.1%	2.99	1.46
90–99	3.3%	3.11	1.76
**Total**	100%	**2.73**	1.52

Further, the Pearson's chi-squared test was applied in order to evaluate if the association between the two variables was a random event. A value under 0.05 was considered statistically significant, which in our case was valid for all the comorbidities studied. After that, the relative risk of exitus for each disease in the SARS-CoV-2 group was computed against that of the general population of Romania, using the most recent statistical data provided by the WHO, from the year 2016. The results showed an increased relative risk for some of the ailments, such as diabetes −6.426 (95% CI, 4.965–8.318), chronic renal disease −4.338 (95% CI, 3.556–5.292), hypertension −3.261 (95% CI, 2.687–3.958), chronic pulmonary disease −2.615 (95% CI, 2.061–3.319) and chronic liver disease −1.577 (95% CI, 1.183–2.104), while for others there was a lower risk, as in the case of coronary heart disease −0.664 (95% CI, 0.581–0.758), cancer −0.515 (95% CI, 0.416–0.637), and stroke −0.468 (95% CI, 0.370–0.593) (Table 8). For all the relative risk values above mentioned, the *p*-value was <0.05. The calculated odds ratios also supported the previously described findings (Table 8).

**Table 8:** Relative risk, odds ratio, Pearson's Chi-Squared test for the most severe comorbidity.

**Most severe comorbidity**	**Relative risk**			***p*-value**	**Odds ratio**			***p*-value**	**Pearson's Chi-Squared test**
	**Value**	**95% CI**			**Value**	**95% CI**			
		**Lower**	**Upper**			**Lower**	**Upper**		
Diabetes	6.426	4.965	8.318	<0.05	6.820	5.173	8.990	<0.05	<0.05
Chronic renal disease	4.338	3.556	5.292	<0.05	4.742	3.796	5.925	<0.05	<0.05
Hypertension	3.261	2.687	3.958	<0.05	3.549	2.854	4.414	<0.05	<0.05
Chronic pulmonary disease	2.615	2.061	3.319	<0.05	2.751	2.125	3.561	<0.05	<0.05
Chronic liver disease	1.577	1.183	2.104	<0.05	1.610	1.188	2.184	<0.05	<0.05
Coronary heart disease	0.664	0.581	0.758	<0.05	0.574	0.485	0.680	<0.05	<0.05
Cancer	0.515	0.416	0.637	<0.05	0.464	0.367	0.587	<0.05	<0.05
Stroke	0.468	0.370	0.593	<0.05	0.423	0.328	0.546	<0.05	<0.05

For the next step, the COVID-19 group was divided into 3 categories, according to the relative risk, as following: for the diseases that had a relative risk over 3 it was considered that the coronavirus had a high impact on mortality, the ones with a relative risk between 1 and 3 were considered medium impact, while those below 1 were considered low impact. In order to determine the negative influence of SARS-CoV-2 over these comorbidities, the attributable risk was calculated for each of them, together with the percentage that could have been prevented if the infection had not occurred (attributable risk percent). Lastly, the mean age for each group was also determined and added into Table 9. The average attributable risk (AR) for the COVID-19 high impact group was 0.219 (95% CI, 0.201–0.236), with an AR% of 75.7% (95% CI, 69.6–81.8%) and a mean age of 66.0. For the COVID-19 medium impact group, the AR was 0.068 (95% CI, 0.051–0.084), with an AR% of 51.4% (95% CI, 38.6–64.2%) and a mean age of 64.8, whereas for the low impact group, the AR was −0.285 (95% CI, −0.317– −0.253), AR% was −74.3% (95% CI, −82.8%– −65.9%) and a mean age of 72.0. It can be observed that the mean age of death was lower in the first two risk categories, compared to the low impact one. All the obtained results can be found in Table 9.

**Table 9:** COVID-19 influence on comorbidities.

**Most severe comorbidity**	**Freq**.	**study group**	**Rom. Pop**.	**Relative risk (95% CI)**	**Attributable risk (95% CI)**	**Attributable risk % (95% CI)**	**Mean age (Std. Dev.)**
COVID-19 high impact	**235**	**28.9%**	**7.0%**	**4.119** (3.694–4.592)	**0.219** (0.201–0.236)	**75.7%** (69.6%−81.8%)	**66.0** (13.32)
Diabetes	55	6.8%	1.1%	6.426 (4.965–8.318)	0.057 (0.05–0.064)	84.4% (74.0%−94.9%)	64.5 (11.54)
Chronic renal disease	88	10.8%	2.5%	4.338 (3.556–5.292)	0.083 (0.072–0.094)	76.9% (67.0%−86.9%)	68.1 (13.47)
Hypertension	92	11.3%	3.5%	3.261 (2.687–3.958)	0.078 (0.066–0.091)	69.3% (58.2%−80.5%)	65.0 (13.82)
COVID-19 medium impact	**107**	**13.1%**	**6.4%**	**2.058** (1.724–2.457)	**0.068** (0.051–0.084)	**51.4%** (38.6%−64.2%)	**64.8** (12.92)
Chronic pulmonary disease	63	7.7%	3.0%	2.615 (2.061–3.319)	0.048 (0.036–0.059)	61.8% (46.7%−76.9%)	67.4 (12.02)
Chronic liver disease	44	5.4%	3.4%	1.577 (1.183–2.104)	0.020 (0.036–0.059)	36.6% (13.4%−59.8%)	61.2 (13.42)
COVID-19 low impact	**312**	**38.3%**	**66.8%**	**0.574** (0.526–0.626)	**−0.285** (−0.317- −0.253)	**−74.3%** (−82.8%- −65.9%)	**72.0** (12.92)
Coronary heart disease	171	21.0%	31.7%	0.664 (0.581–0.758)	−0.107 (−0.139- −0.075)	−50.7% (−65.9%- −35.5%)	73.0 (12.82)
Cancer	77	9.5%	18.4%	0.515 (0.416–0.637)	−0.089 (−0.116- −0.063)	−94.3% (−122.5%- −66.1%)	68.6 (10.15)
Stroke	64	7.9%	16.8%	0.468 (0.370–0.593)	−0.089 (−0.115- −0.064)	−113.5% (−146.1%- −80.8%)	73.4 (9.89)
No & other comorbidities	**160**	**19.7%**	**16.0%**	**–**	**–**	**–**	**66.6** (16.18)
No comorbidities*	21	2.6%	3.8%	–	–	100%	59.0 (17.56)
Other comorbidities**	139	17.1%	16.0%	–	–	–	67.7 (15.72)
Total study group	**814**	100.0%	100.0%	–	–	–	**68.3** (13.61)

* *Assumed all deaths with no comorbidities were due to covid-19*.

** *Other comorbidities were not statistically significant, so were removed.*.

In addition, for the patients with no comorbidities, the cause of death was attributed entirely to the SARS-CoV-2 infection. The group with other comorbidities than the ones studied were not taken into consideration due to the fact that the *p*-value was above 0.05.

## Discussion

Most of the results obtained in this study were in accordance with previous research regarding the SARS-CoV-2 infection. Pertaining to the gender, the findings showed that more deaths occurred amongst men (61.4 vs. 38.6%), in comparison to the deaths in the general population, which seemed to be more balanced and more increased for women (48.5 vs. 51.5%). A prior retrospective study, conducted on 168 patients, also revealed that men were more predisposed to severe outcomes, such as death (12.8 vs. 7.3%), in comparison to women and were more prone to develop critical illness. This might be explained by the fact that the male gender seemed to be more at risk when associating comorbidities, than women (OR = 3.824, 95% CI = 1.279–11.435 vs. OR = 2.992, 95% CI = 0.937–9.558) (50). Similar outcomes have been observed in the case of MERS-CoV and SARS-CoV infections (51, 52). The mechanism why men are more prevalent in the deceased group is not clear, but one possible explanation could be attributed to steroid hormones and X chromosome genes, both of which were previously shown to regulate the immune response in viral infections (53, 54). Another cause that should be considered is the fact that men are less likely to seek early medical consultations for the common diseases, which could have led to more severe states of their underlying conditions (55).

Regarding age, 77.6% of the studied group were over 60 years old, which is in alignment with reported data from China, where 80% of deaths occurred in the 60+ age group (56). Furthermore, the mean age of death in the study group was 68.26 y (SD = 13.609), with a mean age for females of 70.2 y and for males of 67.1 y. In comparison, the average life expectancy provided by the WHO for Romania was 75.2 y, 71.6 y for males, and 79.0 for females (47). Perhaps the lower means could be explained by the coronavirus infection in Romania, however it should be noted that the WHO life expectancy data is from 2016, therefore it refers to the population born in that year. For a more accurate comparison, previous information on life expectancy should be used, altogether with proper adjustments for medical advancements regarding diagnostics and treatment. For more thorough insights, a separate multinational study should be conducted on this matter.

The number of comorbidities analysis showed a mean number of 2.73 (SD=1.521), with a cumulative frequency of underlying diseases of 97.4% for at least one preexisting condition and 50.9% for 3 or more. A previous study conducted in the Wuhan province of China on 1,590 confirmed COVID-19 patients discovered that 25.1% of them had at least one comorbidity, while 8.2% had at least two. They also pointed out that the patients with two or more conditions were more likely to have a severe outcome. After comparing these two groups with the patients without comorbidities, they reached an HR of 1.79 (95% CI, 1.16–2.77) for the people with at least one underlying condition and 2.59 (95% CI, 1.61–4.17) for those with two or more (12). These figures suggest that not only is the presence of comorbidities an influencing factor for the outcome, but also their number plays a proportional part in determining the severity of evolution.

Similar to other studies (12), the linear regression analysis showed a low strength of association between the number of comorbidities and age (R squared = 0.035), with a relatively even distribution of comorbidities throughout the age groups. This could suggest that even though some deaths occurred in younger people, they were very likely to have had at least one underlying condition. The most prevalent disease amongst the studied group was hypertension (43.1%), followed by diabetes (33.2%) and coronary heart disease (26.0%). These results were very close to the ones found by another already published article, that showed a prevalence of 48% for hypertension, 31% for diabetes and 24% for coronary heart disease (57). Although this could be an explicable encounter for coronary heart disease, which, as mentioned in the literature review, represents the number one cause of mortality worldwide, the other two have a considerably higher prevalence in the coronavirus affected population. Further on, statistical tests were applied and the results are next to be discussed.

The statistical analysis applied to the most common comorbidities encountered in the study group revealed an increased relative risk for some of the ailments, while others were at a lower risk compared to the general population. To ease understanding, the authors decided to divide the study sample into three categories, based on the relative risk value. The diseases considered at high risk were diabetes RR=6.426 (95% CI, 4.965–8.318), chronic renal disease RR=4.338 (95% CI, 3.556–5.292), and hypertension RR=3.261 (95% CI, 2.687–3.958), the ones that displayed a medium risk were chronic pulmonary disease RR=2.615 (95% CI, 2.061–3.319) and chronic liver disease RR=1.577 (95% CI, 1.183–2.104) and the low risk group included coronary heart disease RR=0.664 (95% CI, 0.581–0.758), cancer RR=0.515 (95% CI, 0.416–0.637), and stroke RR=0.468 (95% CI, 0.370–0.593).

Similar to our findings, other studies have looked into the association between the SARS-CoV-2 infection and the high risk comorbidities found in this article. Several papers conducted meta-analyses on the relationship between diabetes and Covid-19 related mortality, showing that the presence of diabetes mellitus increased these patients' risk of in-hospital deaths (OR=1.90 and RR=2.12, respectively) and also enhanced the severity and disease progression of Covid-19 (OR=2.75 and RR=3.31) (58, 59). Chronic renal disease was also found to negatively influence the outcome and progression of SARS-CoV-2 infection, with prior meta-analyses showing a hazard ratio (HR) of in-hospital death as follows: HR=2.10 for patients with increased baseline serum creatinine; HR=3.97 for increased blood urea nitrogen; HR=1.90 for patients suffering from stage 1 acute kidney injury, HR=3.51 for stage 2 acute kidney injury and HR=4.38 for stage 3 acute kidney injury, respectively (60–63). Hypertension was one of the most studied comorbidities affected by the novel coronavirus, having multiple meta-analyses that focused on identifying the effect it had on the outcome. Results indicated OR values of 3.36 for elevated mortality risk in hypertensive patients in comparison to normotensive and an OR of 2.49 for the occurrence of a severe form of Covid-19 (64, 65).

The mean calculated ages were 66.0 (SD=13.32) for the high risk group, 64.8 (SD=12.92) for the medium risk and 72.0 (SD=12.92) for the low risk sample. Instead of following an ascending trend from the high to the low risk group, the mean age was found to be lowest in the medium risk group. One possible reason that the authors found, at least in the case of chronic liver disease, was that it has a naturally lower average age of death, even in the absence of SARS-CoV-2 infection. A nationwide analysis performed in Brazil between the years 2000 and 2012, which included 265,180 deaths due to cirrhosis, found that people suffering from this condition had a median age at the time of death of 56 years (95% CI, 47–67) (66).

Prior research looked into the reasons why diabetes, hypertension and chronic renal disease consistently displayed a higher mortality rate amongst the people infected with SARS-CoV-2. One of the common traits that these three diseases and SARS-CoV-2 share is the fact that angiotensin-converting enzyme 2 (ACE2) is involved in their pathogenesis. ACE2 can be found in lungs, kidney, blood vessels and intestine. It was observed that SARS-CoV and SARS-CoV-2 used ACE2 in order to attach to their host cells. It is also well-known that higher levels of ACE2 can be detected in diabetes, hypertension and chronic renal disease patients, who can benefit from ACE2 inhibitor therapy (67, 68). Further research on this matter needs to be undertaken in order to reveal the exact mechanisms that cause these afflictions to be more at risk.

Finally, the authors calculated the attributable risk percentage (AR %) for the infected group against the general population for each comorbidity. The scope was to assess the percentage of deaths that could have been avoided if the SARS-CoV-2 infection had not occurred, for the diseases studied. The results were AR% = 75.7% (95% CI, 69.6–81.8%) for the high risk group, AR% = 51.4% (95% CI, 38.6–64.2%) for the medium risk one and AR% = −74.3% (95% CI, −82.8–−65.9%) for the low risk group. The respective values for each affliction can be found in Table 9. Assuming that the risk that can be attributed to the SARS-CoV-2 infection in the case of people with no comorbidities was 100%, the authors then multiplied the AR% value with the percentages that represented each disease in the high and medium risk from the study group, thus obtaining the proportion of deaths directly caused by COVID-19 in these particular categories. The results were 21.9% (95% CI, 20.1–23.6%) out of 28.9% for the high risk group, 6.8% (95% CI, 5.1–8.4%) out of 1–3.1% for the medium risk group and 2.6% out of 2.6% for the group with no comorbidities. This led to a total of 31.2% (95% CI, 27.8–34.7% out of 44.6%, proportion of deaths that could have been directly caused by the SARS-CoV-2 infection. Furthermore, even though the patients in the last category (38.3%) were considered low risk and were statistically more likely to die because of their underlying condition, more information would be required in order to make a precise assessment of the proportion that could have been directly attributed to SARS-CoV-2 infection. Moreover, in the case of people with other comorbidities (17.1%), a separate study should be conducted, that would take into consideration all of the particularities of the diseases found in that group.

### Clinical Implications

This study has shown that in Romania, the number and type of comorbidities had an important contribution to the outcome of SARS-CoV-2 infection. After taking into consideration all of the aspects and because this paper comes as an additional confirmation of prior studies, one of the most important recommendations for clinical practice would be to offer extra protection to people that have certain types of underlying conditions. Diabetes, end stage renal disease and hypertension were shown to be at high risk when targeted by the novel coronavirus, while chronic pulmonary diseases (COPD and asthma) and chronic liver diseases (in particular cirrhosis) were moderately impacted. All the patients affected by any of these afflictions and especially those at high risk should be closely monitored by their physicians and in the eventuality of SARS-CoV-2 infection, they should present to a hospital in order to be immediately tested. Furthermore, when possible, they should be sheltered and if that desiderate cannot be achieved, they should at least be informed about their situation and the additional precautions they should be taking to protect themselves. Nonetheless their underlying condition should also be monitored and it should be kept under control with an adequate treatment.

In a study published in 2013, the outcome of 91,605 diabetic people was analyzed after the flu vaccination. Scientists then discovered a significant decrease, amongst these patients, in influenza and pneumonia incidence, of up to 43% for people under 65 years old and 55% for those over 65 (69). Taking into consideration the fact that COVID-19 has similar symptoms to influenza (fever, cough, and myalgia) and pneumonia, pneumococcal and flu vaccines could prove of great use in the colder seasons, not only for preventing these afflictions, but also for reducing the potential confusion between them. This matter could prove of great importance especially for people that are more at risk, such as those with the abovementioned comorbidities and the elderly. Lastly, the authors consider that the people in the high and medium risk groups should also be prioritized for future vaccination programs against SARS-CoV-2 in order to attempt to decrease mortality and hospitalization costs. This point is also supported by two official documents released by the WHO and the UK Government. Amongst the categories of people that are considered for vaccine prioritization in the two papers, those who are over 65 and/or suffering from diabetes, chronic kidney disease and chronic pulmonary disease have been included (70, 71).

Another important recommendation that emerged from this study would be for the people in the low risk category. They should be advised to respect their regular follow ups, according to their specialist and should be monitored for their underlying condition as usual. As the study has shown, for this category, the greatest risk is represented by their most severe illness, rather than the SARS-CoV-2 infection, in which case, missing doctors' appointments together with the natural progression of the disease could lead to a premature death. Amongst cancer patients, a recent study calculated an excess of 6,270 deaths in England and 33,890 in the USA, in 1 year due to missed or postponed medical appointments during the pandemic (72). For epidemiological research, the risk factors for each comorbidity could help model the potential fatality rates of SARS-CoV-2, within a given population, based upon the prevalence of these comorbidities.

### Study Limitations

One of the limitations of this study was the lack of information about the people that have been infected and survived in Romania and as a result, the comparison between these two groups was not possible. However, the study was repurposed and the data was replaced with that of the deaths amongst the general population of Romania. This led to another constraint, given the fact that the most recent data available on the WHO website was from 2016. Furthermore, because of outbreaks in certain regions, some patients' information was incompletely reported, leading to a reduction in the study sample. Also, because this was a retrospective study and the data was collected from multiple sources, the accuracy of the information provided did not fall in the authors' responsibility. In addition to those mentioned above, the results discussed in this paper best reflect the population of Romania, as in other countries prevalence rates and therapeutic approaches may vary for the studied comorbidities and therefore, different outcomes could be observed.

Another limitation of this study was the lack of information regarding the initial clinical data at presentation, such as the level of hypoxia, the inflammatory markers and the severity of the disease, which could have offered a better insight on the impact of SARS-CoV-2 infection on the outcome.

### Future Considerations

Another larger, ideally multicenter study comparing both the deceased and the survival group amongst those infected with SARS-CoV-2 would be beneficial in order to further identify preventable causes that led to a worse outcome. This study should also include details regarding laboratory parameters as well as radiological information. Additional analysis on the hospitalization rates for each comorbidity could help hospital capacity planning and preventing severe consequences. Moreover, future research should focus their efforts on developing therapeutic protocols that would improve the survival rates or vaccines to prevent infection.

## Data Availability Statement

Publicly available datasets were analyzed in this study. This data can be found here: http://www.ms.ro/comunicate/.

## Author Contributions

MB, RT, and DT: conceptualization. MB, RT, DT, and DC: methodology. RT and DT: investigation. NS: resources and funding acquisition. MB and DT: writing—original draft preparation. DC: review, editing, and supervision. DT: project administration. All authors contributed to the article and approved the submitted version.

## Conflict of Interest

The authors declare that the research was conducted in the absence of any commercial or financial relationships that could be construed as a potential conflict of interest.
